# Factors Associated with Midterm Visual Field Variability in Patients with Stable Glaucoma

**DOI:** 10.1155/2019/2013160

**Published:** 2019-12-09

**Authors:** Maria Emília V. Guimarães, Carolina P. B. Gracitelli, Syril Dorairaj, Fábio N. Kanadani, Tiago S. Prata

**Affiliations:** ^1^Department of Ophthalmology, Instituto de Olhos Ciências Médicas, Belo Horizonte, Brazil; ^2^Glaucoma Unit, Hospital Medicina Dos Olhos, Osasco, Brazil; ^3^Glaucoma Service, Department of Ophthalmology and Visual Science, Federal University of São Paulo, São Paulo, Brazil; ^4^Glaucoma Unit, Ver Mais Oftalmologia, Vinhedo, Brazil; ^5^Department of Ophthalmology, Mayo Clinic, Jacksonville, FL, USA; ^6^Department of Ophthalmology, Glaucoma Service, Hospital Oftalmológico de Sorocaba—BOS, Sorocaba, Brazil

## Abstract

**Purpose:**

To evaluate factors associated with midterm visual field (VF) variability in stable glaucoma patients in Brazil.

**Methods:**

This retrospective observational study included 59 eyes of 39 stable glaucoma patients. Baseline data assessed were age, gender, educational level, intraocular pressure (IOP), central corneal thickness, best-corrected visual acuity, spherical equivalent, number of hypotensive eye drops, type of glaucoma, number of VFs performed, follow-up in years, lens status, visual field index (VFI) values from the last 5 VF (standard automated perimetry (SAP)) tests, the presence or absence of central scotoma in the VF test, and the level of glaucomatous damage according to the VF mean deviation (MD) index of the last VFs. The 5 latest VFI scores were used to calculate the mean, the standard deviation (SD), and the coefficient of variation (CV). We divided the eyes into 2 groups, being group 1 comprised by the 29 eyes presenting the lowest CV values and group 2 comprised by the 30 eyes presenting the highest CV values. GEE models were used to compare the CV and demographic and clinical parameters of all participants.

**Results:**

Mean age of all subjects was 65.8 ± 10.1 years. 54.0% were women. Average SAP MD values for groups 1 and 2 were −2.8 ± 3.1 dB and −6.2 ± 4.1 dB, respectively (*P*=0.006). Average SAP VFI values for groups 1 and 2 were 95.6 ± 5.9% and 85.9 ± 11.3%, respectively (*P*=0.002). There was a statistically significant association between CV and SAP MD values (*P*=0.006). A worse SAP MD and VFI were associated with a higher CV. In addition, even adjusting for potential confounding factors (age and level of education), the association between CV and the SAP MD and between CV and VFI remained significant (*P* ≤ 0.010).

**Conclusion:**

Glaucomatous patients with worse VF sensitivity scores (both MD and VFI indices) present higher VF test variability.

## 1. Introduction

Glaucoma is an optic neuropathy characterized by progressive loss of retinal ganglion cells associated with structural changes to the retinal nerve fiber layer (RNFL) and optic nerve head (ONH). Loss of visual function in glaucoma is generally irreversible, and without adequate treatment, the disease can progress to disability and blindness [1, 2].

Standard automated perimetry (SAP) remains the reference test for assessment of functional loss in glaucoma and is still the most widely used method to detect progression of visual field (VF) damage [3]. However, SAP is disadvantaged from considerable test-retest variability [4, 5, 6]. Such variability can hinder detection of change, as detection of progression depends on the ability to separate true change (the signal) from test-retest variability (the noise) [7, 8, 9]. In the presence of large test-retest variability, significant changes can be missed and lead to delayed initiation or escalation of treatment [3].

In this context, the ability to know which patients are stable and which one is progressing is the most important cornerstone to early diagnosis and proper treatment of these patients with glaucoma. Many investigations have shown that intraindividual variability of the differential light threshold is considerable and that this variability is particularly large in glaucomatous fields [7, 8, 10–17]. The variability is not uniform and can also be influenced by several factors already described, such as patient's age, race, alertness, visual acuity, refraction, motivation and instruction of the patient, glaucoma disease stage [18, 19], and technician performance [20].

Therefore, the purpose of this study was to evaluate the factors associated with medium term VF test variability in stable glaucoma patients in Brazil.

## 2. Methods

This was a retrospective observational study including 59 eyes of 39 stable glaucoma patients from Glaucoma Unit of Hospital Medicina dos Olhos, Osasco, São Paulo, Brazil. Patient informed consent was not required due to the observational nature of the study where all information came from medical chart review, with no intervention or deliberate modification of biologic, physiologic, psychological, or social variables. All study methods adhered to the tenets of the Declaration of Helsinki for research involving human subjects [21].

At each visit, subjects underwent comprehensive ophthalmologic examination, including best-corrected visual acuity (BCVA, logMAR), spherical equivalent (EE), intraocular pressure measurement (Goldmann applanation tonometry), gonioscopy, slit-lamp biomicroscopy, dilated funduscopic examination, central corneal thickness (CCT), and SAP using the Swedish interactive threshold algorithm (SITA Standard 24-2).

Key inclusion criteria were ≥6 stable VF tests and ≥3 years of follow-up without any changes on the current medical regimen. All first VF test for all patients were excluded due to learning effect. Subjects were excluded if they had any corneal, retinal, or orbital diseases, refractive error greater than  ±4 diopters spherical equivalent, and strabismus. Patients who had undergone medication changes during the study period and who had repeatable progression on 2 consecutive VFs were also excluded.

Patients were diagnosed with glaucoma if they had at least 3 repeatable, consecutive, abnormal VF test results that were defined as a pattern standard deviation outside the 95% normal confidence limits or a glaucoma hemifield test result outside the normal limits and matching the appearance of the optic disc. Patients were also considered to have stable glaucoma if they had nonprogressive VF results (both trend and event analyses) and absence of anatomical changes in the retinography and fundus examination. Nonprogressive VF results were defined as an absence of (1) a new scotoma, defined as 2 adjacent points in a previously normal area, at the 0.01 probability level on the pattern deviation plot, or one point within the central 10° that declined by ≥10 dB; (2) expansion of existing scotoma, defined as 2 contiguous points adjacent to an existing scotoma that declined by ≥10 dB; and (3) deepening of an existing scotoma, defined as 2 points in an existing scotoma that declined by ≥10 dB.

All VFs evaluated had been performed using 24-2 Swedish interactive threshold algorithm (Humphrey Field Analyzer II, Carl Zeiss Meditec, Dublin, USA). VFs with more than 33% fixation losses or false-negative errors, or more than 15% false-positive errors, were excluded. VFs presenting a learning effect (i.e., initial tests showing consistent improvement on VF indices) were also excluded. VFs were also reviewed for the following artifacts: fatigue effects, inappropriate fixation, eyelid and rim artifacts, and evidence that the VF results were caused by a disease other than glaucoma and inattention.

Baseline data assessed were age, gender, and educational level (illiterate, elementary school, high school, and incomplete or complete college). We also assessed the number of hypotensive eye drops, type of glaucoma (primary open-angle glaucoma (POAG), primary angle-closure glaucoma (PACG), or normal tension glaucoma (NTG)), number of VFs performed, follow-up time (in years), lens status (phakic or not), and visual field index (VFI) values from the latest 5 VF tests. We considered the presence or absence of central scotoma and also the level of glaucomatous damage according to the mean deviation (MD) index of the last VF [22]. Early glaucoma was considered if MD ≥−6 dB, moderate if MD between −6 dB and −12.00 dB, advanced if MD between −12.01 dB and −20.00 dB, and severe if MD was ≤−20.00 dB [22].

We used the 5 latest VFI scores to calculate the mean, the standard deviation (SD), and the coefficient of variation (CV). Eyes were then divided into 2 groups, being group 1 comprised by the 29 eyes presenting the lowest CV values and group 2 comprised by the 30 eyes presenting the highest CV values.

### 2.1. Statistical Analysis

Descriptive statistics included mean and standard deviation (SD) for normally distributed variables and median and interquartile ranges for nonnormally distributed variables. Normality assumption was assessed by inspection of histograms and using Shapiro–Wilk tests. Student's *t*-tests were used for group comparison for normally distributed variables and Wilcoxon rank-sum test for continuous nonnormally distributed variables.

Considering that both eyes of the subjects were included in this analysis and that both eyes in a subject would be expected to have some degree of intercorrelation with respect to the results, the generalized estimating equation (GEE) was used to adjust the intereye correlations [23]. After adjusting for within-patient intereye correlations, GEE models were used to compare the CV and demographic and clinical parameters of all participants [23]. Multivariable linear regression model was also created to evaluate the effect of potentially confounding socioeconomic on the association between CV and clinical parameters.

All statistical analyses were performed using commercially available software Stata®, version 13 (StataCorp LP, College Station, Texas, USA). The alpha level (type I error) was set at 0.05.

## 3. Results

Mean age of all subjects was 65.8 ± 10.1 years, ranging from 45 to 84 years. 54.0% were women.  Table 1 shows clinical and demographic characteristics of the sample. Average SAP MD values for groups 1 and 2 were −2.8  ±  3.1 dB and −6.2  ±  4.1 dB, respectively (*P*=0.006). Average SAP VFI values for groups 1 and 2 were 95.6 ± 5.9% and 85.9 ± 11.3%, respectively (*P*=0.002).

**Figure 1:**
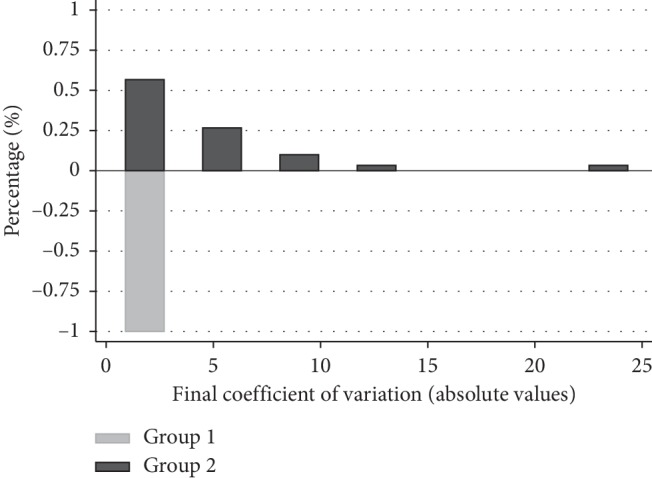
Bar graph showing the distribution of the coefficient of variability (absolute values) in the two groups.

**Figure 2:**
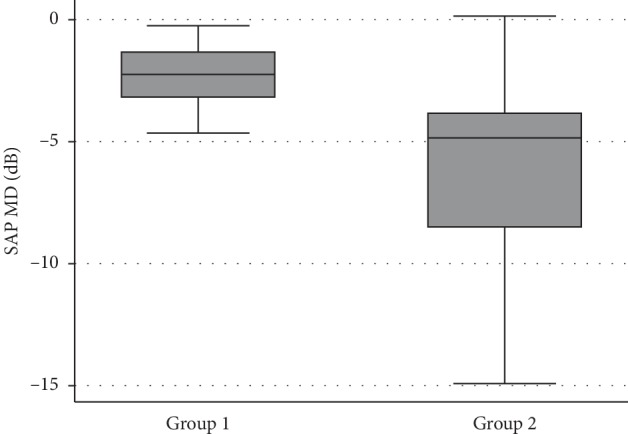
Boxplots showing the distribution of the SAP MD in the two groups.

**Figure 3:**
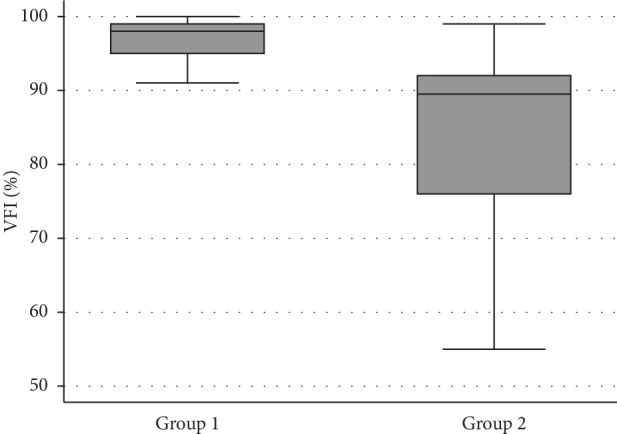
Boxplots showing the distribution of the VFI in the two groups.

**Figure 4:**
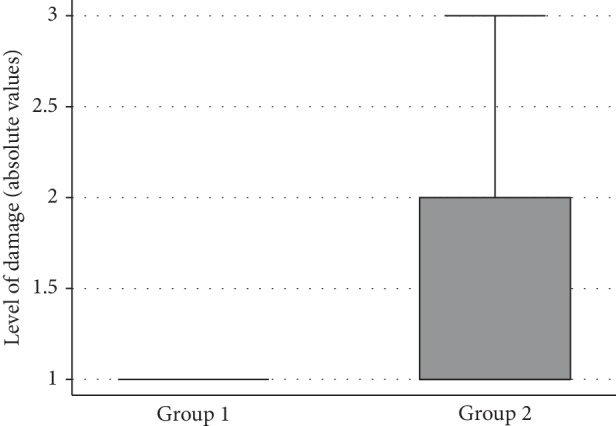
Boxplots showing the distribution of the level of damage in the two groups.

Subjects from group 2 had significantly greater CV compared with group 1 (1.11 and 5.01, respectively, *P* < 0.001). The distribution of CV is shown for the two groups in Figure 1. Those from group 2 also had on average, worse results for SAP MD (*P* < 0.001), VFI (*P* < 0.001), and level of damage (*P* < 0.001) compared with patients from group 1 (Table 1, Figures 2–4).

**Figure 5:**
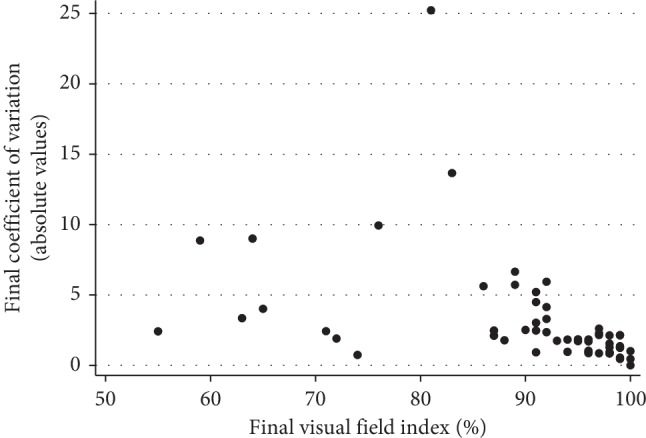
A scatter plot showing the association between the final coefficient of variation and the VFI index in all subjects.

There was a statistically significant association between the CV and the SAP MD (*P*=0.006). A worse SAP MD was associated with a higher CV. There was also a significant association between the CV and the VFI from SAP (*P*=0.003). A worse VFI results were associated with higher CV (Figure 5). In addition, even adjusting for potential confounding factors (age and level of education), the association between the CV and the SAP MD and between CV and the VFI from SAP remains statistically significant (*P*=0.010 and *P*=0.001, respectively). Finally, there was also a significant association between CV values and the type of glaucoma (*P*=0.021). Primary open-angle glaucoma was associated with higher CV values. However, when potential confounding factors (age and level of education) were included in the analysis, the association between CV and the type of glaucoma was not significant (*P*=0.094).

The association between race and VF variability was not investigated in this study due to the small sample size (only two self-reported African descendants).

## 4. Discussion

The present study found that patients' worse SAP MD and VFI were associated with a higher CV in the VF test in patients with stable glaucoma. To our knowledge, this is the first study to assess factors associated with midterm VF variability in patients with stable glaucoma.

The variability in SAP can be influenced by several factors [19, 20, 24–28]. It is important to consider the variability inherent in psychophysical procedures since SAP is a subjective test that aims to measure a sensitivity threshold in a living organism; therefore, test results are prone to variability [20] and some variability will always be present when testing human vision [19]. There is patient-related variability such as motivation, instruction, and cooperation [20, 24]. The fixation loss rate can be high with patients' distraction and fatigue [24]. High false-positive rates usually occur due to lack of understanding of the test or anxiety from the patient [24]. There is also intrinsic physiological variability present in diseased eyes [19]; for example, in advanced cases of glaucoma, it may reflect the larger fluctuation in response to the borders of pre-existing scotomas [24]. Depressed areas of the VF, manifesting as locations with reduced threshold sensitivities, are accompanied by increased test-retest variability [25–28].

The present study is in agreement with previous ones. For example, Blumenthal et al. [19] in a prospective study evaluated which factors contributed to the variability in VF. The most important factors analyzed in their study were age, diagnosis, severity, eccentricity, pupil size, location in the VF, and between-subject variation. They calculated the test-retest variability (TRV) for 41 controls, 10 suspects, and 35 stable glaucoma patients. Also, they concluded that defect severity at any given location was the single largest factor associated with increased TRV. Severity was found to have a much larger effect on TRV than the diagnosis status of the individual tested, while most of the factors in their model exerted a statistically significant measurable effect on TRV, and they were found to contribute modestly to the overall variability noted. Over 70% of the variability remained unaccounted for [19]. In the present study, we did not evaluate the location of VF test and the eccentricity.

The SAP measurements are known to be quite variable, especially when VF damage becomes evident [4, 29–31]. In the present study, there is a statistically significant association between the CV and the SAP MD (*P*=0.006) and the VFI results (*P*=0.003). A worse SAP MD and VFI were associated with a higher CV. In addition, even adjusting for potential confounding factors (age and level of education), the association between the CV and the SAP MD and between CV and the VFI from SAP remains statically significant (*P*=0.010 and *P*=0.001, respectively).

In the current study, the association between race and VF variability was not investigated due to the small sample size (two self-reported African descendants). The term race is complex and can represent a great biodiversity of cultural, geographic, biological, and socioeconomic characteristics [32, 33]. In addition, the Brazilian population is very mixed, so it is difficult to determine the races. Some authors have reported in other studies a relationship between race and reproducibility of the visual field. According to Gracitelli et al. [3], African descent (AD) was significant associated with higher CV. In their study, patients of African descent with glaucoma showed increased VF variability compared with those of European descent (ED) even after adjustment for socioeconomic or educational background [3]. The authors followed a cohort of 236 eyes of 173 individuals of ED and 235 eyes of 171 individuals of AD over time. In their study, the TRV was approximately 30% larger in individuals of AD vs. those of ED. Worse VF damage was also associated with increased variability in their study.

Other factors such as cognitive level may also influence VF variability. A recent study by Diniz-Filho et al. [34] suggested that differences in cognitive level could be related to levels of VF variability over time. The authors followed a cohort of 115 patients with glaucoma and suspects over time and assessed cognitive level with the Montreal Cognitive Assessment (MoCA) test. They found a 5-point decline in the MoCA test scores associated with a 0.18 dB increase in the standard deviation of the SAP MD residues, indicating increased VF variability (95% CI, 0, 06 −0.30, *P*=0.003) [34]. They concluded that cognitive decline was associated with increased VF variability during follow-up [34]. We were not able to assess overall cognitive status in the subjects enrolled in the current study.

In our study, there was no association between average IOP mmHg and VF variability (*P*=0.094). According to Gardiner et al. [35] who studied the seasonal variations in climate in 6 geographic zones of the United Statesand also evaluated the seasonal peaks in IOP and the VF sensitivity. There was no evidence of any causal relation between the seasonal peaks in IOP and VF sensitivity. Although IOP also exhibits seasonal variations, there was no evidence of a causal relation [35]. In our study, we did not evaluate the effect of the season on the VF variability. In Brazil, there is no marked seasonal variation in the climate as in the United States.

The current study included patients from 45 to 84 years, and there was no association between the age and VF variability (*P*=0.855). In agreement with Blumenthal and colleagues [19], the age contribution to the VRT was 0.1% for the standard VF (*P*=0.73) and the age of their patients ranged from 26 to 80 years [19].

There was no association between BCVA, logMAR and VF variability in our study. In group 1, the mean BCVA, logMAR was 0.08 ± 0.15 and in group 2, 0.11 ± 0.13 (*P*=0.385). According to Matsuura et al. [36], reproducibility of the VF appreciably becomes worse as VA decreases, in particular, when logMAR VA is >0.5. Furthermore, VA was not significantly related to well-known VF reliability indices (fixation loss, false-positive, and false-negative) [36].

The main idea of this study was to alert us that although fundamental in monitoring the patient with glaucoma, perimetry remains patient-dependent. The differences inherent to each patient imply directly the ability to detect progression in the exam. Thus, both the variabilities presented by the patient and the progression rate of each patient make it more or less difficult to distinguish who is progressing from those who are not. Variables associated with VF loss and VF variability may help identify patients who need greater clinical scrutiny. Variability obscures the identification of true glaucomatous progression in VF and makes detection of progression challenging [37]. Therefore, reducing variability is particularly important [30] as the change in a glaucomatous VF must be greater than the noise about the measurement before it becomes statistically distinguishable [25, 28]. Most of the studies refer to the European and North American population. Studies on this subject are lacking in the South American population (Brazil), which is also one of the intentions of our study.

It is important to address some specific points of this study. First, it was a small sample size and a cross-sectional study; however, it was the first study to assess factors associated with midterm VF variability in patients with stable glaucoma in South American population (Brazil). Second, some patients may progress by structural parameters like in the optic coherence tomography (OCT), which was not evaluated in this present study. The OCT could identify progression in some eyes that were not identified as a progressor by the VF test and it is one limitation of our study. This would be more expected in eyes with initial disease, different from the population of our study. The use of the conventional OCT assessment would not apply to many of the eyes of this study because a significant part of these patients already had advanced glaucoma.

In conclusion, glaucomatous patients with worse VF sensitivity scores (both MD and VFI indices) present higher VF test variability. These findings may influence the detection of functional glaucomatous progression in these patients and should be considered while interpreting their VF test results over time. We suggest a higher test frequency for these patients and that any VF change should be reconfirmed to increase the likelihood that the observed change is truly due to progressing glaucoma. Delayed detection of progression and possible delayed intervention could explain in part the high rate of visual impairment in these patients.

## Figures and Tables

**Table 1:** Demographic and clinical ophthalmological characteristics in group 1 and group 2.

	Group 1 (*N* = 20)	Group 2 (*N* = 19)	*P* value
Age, years^a^	65.5 ± 10.3	66.1 ± 9.9	0.855^c^
Gender, %			0.152^e^
Female	13 (62%)	8 (38%)	
Male	7 (39%)	11 (61%)	
Ancestry, %			0.136^e^
African	0 (0%)	2 (11%)	
MD SAP, dB^b^	−1.11 (0.00 to −1.84)	−5.01 (−1.90 to −25.23)	<0.001^d^
Final coefficient of variability^b^	1.11 (0.00 to 1.84)	5.01 (1.90 to 25.23)	<0.001^d^
Number of visual fields	8.48 ± 1.90	8.60 ± 1.83	0.810^c^
Average IOP, mmHg^a^	13.45 ± 3.00	12.20 ± 2.62	0.094^c^
VFI, %^b^	95.93 (74–100)	84.53 (55–99)	<0.001^d^
CCT, *µ*m^a^	507.48 ± 38.65	508.32 ± 38.04	0.936^c^
Spherical equivalent^a^	−0.30 ± 1.89	−0.26 ± 1.47	0.933^c^
BCVA, logMAR^a^	0.08 ± 0.15	0.11 ± 0.13	0.385^c^
Number of medication	1.62 ± 0.78	1.30 ± 0.88	0.143^c^
Follow-up time	5.6 ± 1.23	5.0 ± 1.53	0.184^c^
Level of damage, % (advanced)	1 (5%)	6 (32%)	0.022^e^
Central scotoma, % (yes)	2 (10%)	16 (84%)	0.002^e^
Type of glaucoma, % (POAG)	12 (60%)	17 (90%)	0.200^e^
Pseudophakic, % (yes)	9 (45%)	7 (37%)	0.081^e^
Educational level, % (at least high school degree)^a^	20 (100%)	18 (95%)	0.524^e^

MD = mean deviation; dB = decibels; IOP = intraocular pressure; VFI = visual field index; CCT = central cornea thickness; and BCVA = best-corrected visual acuity. ^a^Mean ± SD. ^b^Mean (range). ^c^*t*-test. ^d^Wilcoxon rank-sum test. ^e^Pearson's chi-squared test.

## Data Availability

The excel data used to support the findings of this study are included within the supplementary information file(s).
